# Occupational Formulation: A Scoping Review of Its Development and Use

**DOI:** 10.1155/oti/2011857

**Published:** 2026-06-19

**Authors:** Lorrae Mynard, Ellie Fossey, Genevieve Pepin, Louise Farnworth

**Affiliations:** ^1^ Department of Occupational Therapy, Monash University, Frankston, Victoria, Australia, monash.ac.za; ^2^ Occupational Science and Therapy, Deakin University, Geelong, Victoria, Australia, deakin.edu.au

## Abstract

**Introduction:**

Occupational formulation offers a way to synthesise assessment information to understand a person′s occupational participation and needs. Although increasingly recognised in professional guidelines, there has been limited exploration of its development and application in occupational therapy.

**Methods:**

A scoping review was conducted following Arksey and O′Malley′s framework and PRISMA‐ScR guidelines. Comprehensive searches were undertaken across five databases (AMED, CINAHL, Emcare, PsycInfo and Google Scholar), a global theses repository, and multiple grey literature sources. Inclusion criteria focused on English sources describing the use by occupational therapists of formulation or conceptualisation related to occupational concepts. One hundred and two eligible sources were identified, from which data were extracted, tabulated and synthesised narratively.

**Results:**

The reviewed sources spanned 2002–2025, mainly originating from the United Kingdom and United States, and mental health settings. Sources included some evaluations and practice reflections but limited empirical research. Four strands of occupational formulation development were identified: formulation associated with the Model of Human Occupation, descriptions of the occupational therapy practice process, psychological formulation approaches and bespoke occupational approaches. Identified key features of occupational formulation were as follows: its grounding in occupational theory, inclusion within the practice process between assessment and goal development/therapy planning, collaborative emphasis, narrative approach, provision of a structure and being an ongoing process. Reported purposes included synthesising assessment findings, supporting reasoning, supporting shared understanding and therapy planning, enhancing communication and promoting an occupational focus. Benefits for the person, therapeutic relationship, occupational therapist and team were reported.

**Discussion:**

Occupational formulation is both a process and a written product that can strengthen occupational therapy practice by linking theory to practice, enhancing reasoning and supporting collaborative care. Definitions are proposed to support consistent use in research and practice.

**Conclusion:**

Occupational formulation shows promise for enhancing occupational therapy practice. Further research should evaluate its effectiveness across diverse practice settings and populations.

## 1. Introduction

Occupational therapy involves complex clinical reasoning [1, 2] for linking understanding of occupation to assessment, identification, analysis and prioritisation of occupational needs, and facilitation and evaluation of occupational engagement [3]. However, these unique core skills of occupational therapy are largely invisible or hidden in practice [1, 3]. Recognising this challenge, Thompson [4] asserted that both clinical reasoning and case formulation are needed to identify the focus of occupational therapy in practice, support development of a shared understanding of clients′ situations and enhance their engagement in therapy.

The term ‘occupational formulation’ is increasingly used in occupational therapy, as reflected in professional guidelines in the United Kingdom, including the 2023 Standards of Proficiency for Occupational Therapists [5–7]. In Australia, it has been included in the capability framework for occupational therapists working in mental health since 2023 [8, 9]. In these documents, occupational formulation is only referred to broadly as part of the process of using information gathered from assessments to guide intervention planning in occupational therapy practice. The professional expectations on occupational therapists to implement occupational formulation in practice raise an urgent need to understand its development and practice applications.

The use of case formulation is better established and defined in psychology than occupational therapy [10]. Within psychology, Hass et al. [11] described case formulation as ‘the collection, organisation and interpretation of individual and contextual data to provide a comprehensive picture of clients and their strengths and needs; potential explanations or hypotheses for an individual′s present psychological, interpersonal and behavioural challenges; and possible treatments or interventions’ (pp. 4–5). Described as a bridge between assessment and treatment [12], both the American Psychological Association [13] and British Psychological Society [14] identify case formulation as a core component of clinical expertise. Some authors have asserted that psychological case conceptualisation and formulation are synonymous [11, 15], but the root terms are differentiated in the Oxford dictionary [16]. *Conceptualization* is the act or process of forming an idea in the mind, whereas *formulation* is both the action of creating or preparing something, and material prepared according to a formula.

To extend understanding of formulation within occupational therapy, this review focuses on occupational formulation, meaning the formulation of a person′s situation using an occupational lens. Preliminary searches (Joanna Briggs Institute, Cochrane and EBSCOhost CINAHL Plus) found no prior reviews or primary research on this topic. A scoping review approach was therefore chosen to map concepts and evidence, inform practice and identify knowledge gaps [17, 18]. Specifically, it is aimed at exploring how occupational formulation is understood, its development, evidence base and use in occupational therapy practice.

## 2. Methods

The scoping review design followed the methodological steps described by Arksey and O′Malley [19], as outlined below. Recommendations and guidelines from Levac et al. [20], the Joanna Briggs Institute [21] and the PRISMA Sc‐R checklist [22] also informed its design. A scoping review protocol was developed and uploaded to Figshare [23].

### 2.1. Identifying the Research Question

As occupational formulation is potentially applicable widely within occupational therapy and not limited by population or setting, a broad mapping research question was identified: What is known about occupational formulation, and how is it used in occupational therapy practice?

### 2.2. Identifying Relevant Sources

An academic research librarian was consulted regarding selection of search platforms and strategies. Preliminary searching indicated that limited evidence may exist, so all forms of evidence were included, without date or limitations. The term ‘source’ [21] was selected in recognition that a range of information, not just ‘studies’ might be identified. Key terms used within searches to capture the context and concept of interest were occupational therapy AND formulation OR conceptualization.

Separate searches were conducted by the first author (L.M.) using five databases: OVID AMED (database records 1985–present), OVID APA PsycInfo (database records 1806–present), OVID Emcare (database records 1995–present), EBSCOhost CINAHL Complete (1984–present) and Google Scholar. A thesis search was conducted in ProQuest Dissertations and Theses Global (fulltext database records 1997–present). Based on preliminary searches indicating limited primary research, ‘grey information’ [24] searches were included, using multiple complementary strategies as suggested by Godin et al. [25] to increase the reach of records and decrease the risk of omitting relevant sources. Textbooks of potential relevance were identified in a Google Books search. Further internet searches included three Google searches, one DuckDuckGo search to account for filter bubbles that may affect Google searches and a targeted search within the Model of Human Occupation (MOHO)–Intentional Relationship model (IRM) website (https://moho-irm.uic.edu/). Initial searches were conducted in November–December 2023 and updated in July 2025. All searches were documented in an audit trail (for full search strategies, see supporting information).

Hand searching of authors and forward/backward searching of reference lists of selected sources was conducted by L.M., limited by the earliest date of sources found during primary searches (2002). Where relevant, L.M. contacted authors for further information, with seven providing further material.

### 2.3. Source Selection

Eligibility criteria were iterative [20], and inclusion and exclusion criteria pilot tested for reliability. Inclusion criteria were as follows:

• occupational therapy sources (inclusive of multidisciplinary sources with occupational therapy contribution)
• use of an occupational lens in formulation OR conceptualisation (i.e., occupation‐related concepts or frameworks)
• formulation OR conceptualization applied at an individual level

Exclusion criteria included:

• sources from other disciplines
• broad use of the key terms (e.g., formulation/conceptualisation of an idea/programme/policy)
• formulation at a population level
• psychological formulation by occupational therapists with no connection to occupational concepts
• sources authored by the primary review author (L.M.)
• multimedia or nonwritten sources
• sources in a language other than English for which translation was unavailable

Potential sources identified in database searches were collated and uploaded into EndNote 21, then duplicates removed. Titles and abstracts were first screened in Covidence and potentially relevant sources were retrieved. Next, full texts were screened against the inclusion and exclusion criteria. Screening was conducted independently by L.M. and L.F. at each stage, with reasons for exclusion recorded and discrepancies resolved through discussion. For theses, L.M. undertook initial title screening followed by abstract screening within the repository.

Grey information screening processes were undertaken by L.M. and varied according to the search platform and information available. For internet searches, removal of duplicates and initial screening was integrated with the search process by reviewing titles and the short text underneath, followed by further content review when needed. Those that met inclusion criteria were bookmarked and filed in subfolders named by search strategy [25]. For Google Books searches, the title and short text underneath were reviewed and, for those that appeared to meet inclusion criteria, the *preview* and *search inside* functions used to identify those for full text review. Full text review was conducted by reviewing electronic or hard copies of books. For edited books in which search terms appeared, the whole book was searched and chapters reflecting the target concepts were included. Any uncertainties regarding source relevance were discussed with the authorship team to inform final selection.

**Figure 1:**
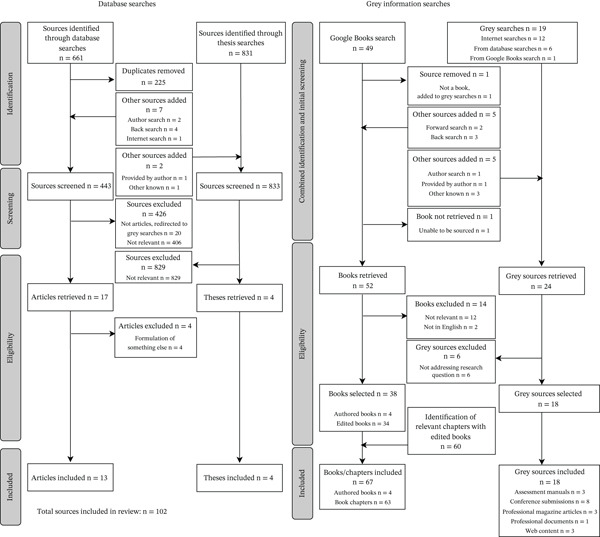
Modified PRISMA flow diagram of source searching, screening and selection.

The results of the searches are reported as a PRISMA flow diagram in Figure 1.

### 2.4. Charting the Data

The first author (L.M.) extracted data, entering it into an Excel spreadsheet. Extraction fields were expanded once source content was inspected and included source characteristics and information relevant to the research objectives (e.g., terminology used and definitions provided). The full list of extraction fields is available within File S1.

### 2.5. Collating, Summarising and Reporting the Results

Descriptive characteristics and presence of key content from sources were tabulated. Narrative synthesis was employed to systematically organise and interpret findings from diverse sources [26]. This involved identifying key concepts, patterns and relationships across the included sources, to reach a comprehensive understanding of the development, description, use of and evidence for occupational formulation.

### 2.6. Informal Consultation

Arksey and O′Malley described consultation as optional to identify additional references and offer insights beyond the literature. Rather than a formal consultation period, liaison with known authors of sources and occupational therapists with experience teaching or using occupational formulation was conducted by L.M. to support identification of additional sources and understanding of key concepts.

## 3. Results

This scoping review sought to explore how occupational formulation is understood and used in occupational therapy practice. A total of 102 sources were identified (13 peer‐reviewed, 4 theses, 67 book chapters and 18 grey sources), mainly originating from the United Kingdom and United States and mental health settings. Sources included some evaluations and practice reflections but limited empirical research. The source characteristics are summarised below, followed by a synthesis of their content describing firstly, the emergence and development of occupational formulation; secondly, the key features of occupational formulation; thirdly, its key purposes; and fourthly, reported benefits of implementing occupational formulation.

### 3.1. Source Characteristics

Table 1 presents the 102 included sources ordered by publication year (2002–2025), summarising their characteristics, the terminology used and the features and purposes of using occupational formulation. A legend is provided in the notes below the table.

**Table 1:** Characteristics and key elements of sources.

	Characteristics				Term/s used			Features						Purpose					
	Country^*^	Content type^*^	Practice area^*^	Objective ^*^	Standalone terms^*^	Stem terms^*^	Focus terms^*^	Incorporates theory/model/framework^*^	Within practice/reasoning process	Developed collaboratively	Incorporates narrative approach	Structured	Ongoing/requiring revision	Synthesises assessment findings	Supports therapeutic reasoning	Supports shared understanding	Supports setting priorities/goals/plans^*^	Communicates reasoning, plans/documented^*^	Offers occupational perspective
**2002**																			
^∗∗^Chapters in *A Model of Human Occupation:* Theory and Application, 3rd edition [27 **]**																			
^∗∗^Forsyth and Kielhofner [28]	GB, US	T	OT	P		C, X	C, S	IM	X	X			X	X	X		T	D	
^∗∗^Kielhofner [29]	GB	T	OT	P		C		IM	I								T		
^∗∗^Kielhofner et al. [30]	US, SE, BE	A	CI	M		F	C	IM	I					I					
^∗∗^Kielhofner et al. [31]	US, DE	A	OT	M		C	C	IM	I										
^∗∗^Kielhofner et al. [32]	US, GB	A	OT	M		C	C	IM	I	X				I			P		
^∗∗^Kielhofner and Forsyth [33]	US, GB	T	OT	P		C	C	IM											
^∗∗^Kielhofner and Forsyth [34]	US, GB	T	OT	M	C	C	C	XM		X				X		X	P, T		
^∗∗^Kielhofner and Forsyth [35]	US, GB	T	OT	P	C, F	T, E	C	IM	X	X							P, T		
**2003**																			
Creek [36]	GB	T	OT	P	PF		S	X	X	X							T		
**2004**																			
Mairs and Bradshaw [37]	GB	T	MH	W	CF			XO	X								T		
**2006**																			
Duncan [38]	GB	T	OT	P	PF				X										
Parkinson et al. [39]	GB, US	A	MH	M	CF			IM	X										
Wimpenny et al. [40]	GB	R	MH	M	CF			IM						X					
**2008**																			
^∗∗^Chapters in *Occupational Therapy and Mental Health*, 4th edition [41]																			
^∗∗^Cole [42]	GB	A	MH	W		C	S	IM	I								T	D	
^∗∗^Creek [43]	GB	T	MH	P	PF				X										
^∗∗^Creek and Bullock [44]	GB	T	MH	P	PF				X								P		
^∗∗^Creek and Bullock [45]	GB	T	MH	P	PF		S	X		X			X						
^∗∗^Chapters in *Cognitive Behavioural Interventions in Physiotherapy and Occupational Therapy* [46]																			
^∗∗^Davidson and Joice [47]	GB	A	MH	W	F			IM, O		X			X	I			T		
^∗∗^Duncan [48]	GB	A	MH	W	F			IM, O											
^∗∗^Chapters in *Model of Human Occupation: Theory and Application* [49]																			
^∗∗^Auzmendia et al. [50]	US, CL, AR	A	OT	M	C			IM											
^∗∗^Forsyth and Kielhofner [51]	GB, US	T	OT	M		C, X	C	XM	X	X							P, T	C, D	
^∗∗^Kielhofner et al. [52]	US, GB, IS, BE	A	CI	M	C			IM											
^∗∗^Kielhofner and Forsyth [53]	US, GB	T	OT	P			C	IM	I	X							P		
^∗∗^Kielhofner and Forsyth [54]	US, GB	T	OT	P		C, F, X	C	XM	X	X			X	X					
^∗∗^Kielhofner and Forsyth [55]	US, GB	T	OT	M		C	C	XM		X				X			T		
Keponen and Launiainen [56]	FI	A	OT	M				IM									P, T		X
Parkinson et al. [57]	GB	A	MH	M	CF	C, F	C	IM				X		X	X	X	P, T	D	
**2009**																			
CIRCLE Collaboration [58]	GB	A	CY	M	CF, C			IM			X			X	X		T		
^∗∗^Chapters in *Skills for Practice in Occupational Therapy* [59]																			
^∗∗^Prior and Duncan [60]	GB	T	OT	P	C				X	X				X		X	P, T		
^∗∗^Taylor and Melton [61]	US, GB	T	OT	M		F	S	XM											
Kielhofner [62]	US	T	OT	M, P		C, X	C	XM	X				X	X			P, T		
Kielhofner et al. [63]	US, GB	T	OT	M		C, X	C	XM	X					X			T		
Mahaffey [64]	US	A	MH	M				IM	X										X
**2010**																			
O′Brien et al. [65]	US	R	CY	M		C	C, S	XM	X								T		
Spearing et al. [66]	GB	A	MH	F	OF			IM, O							X		P		
Turpin and Iwama [67]	AU, CA	T	OT	M		C	C, S	IM	X										
Wimpenny et al. [68]	GB	R	MH	M	CF			IM						I					
**2011**																			
Dora [69]	—	T	MH	F	CF			IM						X	X		P, T		X
Duncan [70]	GB	T	OT	P	PF				X										
Parkinson [71]	GB	T	MH	M	CF, OCF			IM			X	X					P	D	
Parkinson et al. [72]	GB	R	MH	F	CF, OCF			IM	X			X					P	C, D	X
**2012**																			
Bullock [73]	GB	T	OT	F	OF			XO	X			X	X		X		T	C, D	
David and Sojka [74]	GB	A	MH	M	CF			IM											
Martin‐Saez et al. [75]	GB	A	CI	M	OF			IMO	I					I		X			
Thompson [4]	NZ	T	OT	F	CF						I				X	X	P, T	C, D	
Withers et al. [76]	GB	A	CI	W	OF			IM									T		
**2013**																			
Winegardner and Rich [77]	GB	A	CI	W	OF			IM	I						I		T		
**2014**																			
^∗∗^Chapters in *Creek′s Occupational Therapy and Mental Health*, 5th edition [78]																			
^∗∗^Creek [79]	GB	T	MH	P	PF		I	X	X				I				P		
^∗∗^Lee and West [80]	GB	A	OT	W	CF		I	IO	I	X				X		X	P, T		
^∗∗^McCullough [81]	GB	T	MH	P					X	X							P		
Forsyth et al. [82]	GB	T	OT	M	OF	X	C	XM	X					X			T		
Graff [83]	NL	R	MH	W	CF			IM	X	X	X			X			P, T		
**2015**																			
ACHIEVE Alliance [84]	GB	A	CY	M	OF	X	C	IM	X				X	X					
Connell [85]	GB	T	MH	F	CF			IM, O		I				X			T		X
Parkinson et al. [86]	GB	R	MH	W	OCF			IM						X			P		
**2016**																			
Brooks [87]	GB	R	MH	W	OF			IM	X		X		X					D	X
Connell [88]	GB	R	MH	W	F														I
O′Reilly and Johnson [89]	GB	A	MH	W	OF			XM, O											
**2017**																			
Claridge et al. [90]	GB	A	MH	M	OF			IM	X					I					
Inman [91]	GB	R	MH	W	OF ON				X	X	X		X			X	P, T	D	
Jamieson and Parkinson [92]	GB	A	MH	F	CF			XM	I			X		X			P		
O′Brien [93]	US	T	OT	M		C	C, S	IM	X					X	X				
O′Brien et al. [94]	US	T	OT	P		C	C								X				
Parkinson and Jamieson [95]	GB	A	MH	F	CF, OCF		C	IM			X	X						C, D	
Rigby and Wilson [96]	GB	A	MH	W	F			XO		X			X	X			I		
^∗∗^Chapters in *Kielhofner′s Model of Human Occupation:* Theory and Application, 5th edition [97]																			
^∗∗^Cahill et al. [98]	US	A	CY	M				XM	X	X					X				
^∗∗^de las Heras de Pablo et al. [99]	CL, GB, AU	T	OT	M		C		IM											
^∗∗^de las Heras de Pablo et al. [100]	CL, AU, US	A	OA	M		C		IM											
^∗∗^Forsyth [101]	GB	T	OT	P	OF		C	IM	X	X				X					X
^∗∗^Melton et al. [102]	GB, US	A	MH	M	CF			IM								I			
**2018**																			
Brooks and Parkinson [103]	GB	T	OT	F	OF		C	XM	X		X	X						C, D	
**2019**																			
Forsyth et al. [104]	GB	T	OT	M	OF	X	C, S	XM	X								T		
Strong and Rebeiro‐Gruhl [105]	CA	T	MH	P		F	I	IO		X					X	X			
**2020**																			
Duncan [106]	GB	T	OT	P					X										
Graham [107]	GB	R	MH	W	OF			IM		X								C	
**2021**																			
Christie [108]	GB	R	MH	W	OF, ON				X	X						X	X		
Gustaffson et al. [109]	AU	T	OT	P	OF		C	IM	X	X				X			P, T		X
Parkinson and Brooks [110]	GB	T	OT	F	OF			XM	X	X	X	X		X		X	P, T	C, D	X
**2022**																			
Bicker and Holley [111]	AU	A	MH	W	OF			IM											X
Bicker and Holley [112]	AU	A	MH	W	OF			IM			X								X
^∗∗^Chapters in *Creek′s Occupational Therapy and Mental Health*, 6th edition [113]																			
^∗∗^Kemble and Plastow [114]	GB, ZA	T	OT	P	OF		C, I	XM		I	X	X	X	X			P, T	D	
^∗∗^Plastow and Bryant [115]	ZA, GB	T	MH	P		F		I	X	X				X			P, T	C	
^∗∗^Sims [116]	GB	A	MH	W	CF			IM	X	X							P		
Khemthong et al. [117]	TH	A	MH	P	OF			X	X	I	X	X			X				
**2023**																			
Brooks [118]	GB	A	OT	F	OF				X		X	X						D	
Graham [119]	GB	R	MH		OF				X	X	X						P, T		
^∗∗^Chapters in *Therapeutic Reasoning in Occupational Therapy: How to Develop Critical Thinking for Practice* [120]																			
^∗∗^O′Brien [121]	US	T	OT	M		C	C, S	IM	X				X	X	X				
^∗∗^Patnaude [122]	US	T	OT	P	C			XM						X					
^∗∗^Patnaude and O′Brien [123]	US	A	OT	P	C			IM							X		P, T		
^∗∗^Reidy and Andrejow [124]	US	T	OT	P		C	I	IM	X				X	X	X		P		
Taylor and Melton [125]	US, GB	T	OT	M		F	S	XM											
**2024**																			
Forsyth and Brown [126]	GB	T	OT	M	OF	X	C	XM	X					X			P, T		
Parkinson [127]	GB	A	MH	M	OF			IM				X							
^∗∗^Chapters in *Kielhofner′s Model of Human Occupation:* Theory and Application, 6th edition [128]																			
^∗∗^Bowyer et al. [129]	US	A	CY	M		C	S	XM	X				X	X	X				
^∗∗^Fisher et al. [130]	US, GB	A	PR	M		C	S	IM		X				X			P, T		
^∗∗^Melton et al. [131]	GB, US	A	MH	M	CF			IM											
^∗∗^Wolske et al. [132]	US, AU	T	OT	P	C	C	S	XM	X	X	X		X	X	X		P, T		
Turpin et al. [133]	AU, CL, US, CA	T	OT	M	OF			XM	X					X					
**2025**																			
Brooks and Grimwood [134]	UK	T	OT	P	OF				X	X	I				X		X		
Cullen [135]	US	T	CY	F	OF			XM				X			I	X	X		
Popova and Scott [136]	US	T	OT	M		C	S	IM	X	I									
Strong and Rebeiro‐Gruhl [137]	CA	T	MH	P		F	I	IO		X						X			

*Note:* Columns marked with an asterisk use letters to represent specific concepts as indicated here. In all other columns the presence of an element is indicated as implicit (I) or explicit (X). **Country:** Alpha 2 ISO country codes designate country names. **Content type:** theoretical (T), applied (A), and research and evaluation (R). **Practice area:** all areas (OT), mental health (MH), cognitive rehabilitation, (CI) child/youth (CY), older adult (OA), and physical rehabilitation (PR). **Objective:** to describe…formulation/conceptualisation (F), MOHO (M), practice process/theory links (P) and occupational therapy in practice (W). **Standalone terms:** conceptualisation (C), case formulation (CF), formulation (F), occupational formulation (OF), occupational case formulation (OCF), occupational needs formulation (ONF) and problem formulation (PF). **Stem terms:** conceptualisation of… (C), explanation of… (E), formulation of… (F) and theory‐based understanding of… (T). **Focus terms:** client′s (occupational) circumstances/status/situation (C), client′s strengths/abilities/challenges/needs (S) and occupational performance issue/problem (I). **Incorporates theory/model/framework**â€”the first letter represents whether the source indicated use of a theory/model/framework: implied (I) and explicit (X), and the second letter represents whether a specific model was suggested: MOHO (M) and other (O). **Supports selecting priorities/goal setting and therapy planning/provision:** priorities/goal setting (P) and therapy planning/provision (T). **Communicates reasoning, plans/documented:** communicates reasoning/plans (C) and documented (D). The asterisks (∗∗) denotes chapters within an edited book.

Seventy percent of sources (71/102) had an author from the United Kingdom; 70% had an author from the United States (71/102); 8% had an author from Australia (8/102), with authors from another 12 countries also represented. Source type varied, with 13 peer‐reviewed articles including research reports, opinion pieces, case studies, practice or education evaluations. Two thirds (67/102) of sources were books or book chapters, including chapters from sequential editions of edited books [27, 49, 59, 67, 78, 97, 113, 128, 133, 138–147]. The 10 edited books are shaded in gray in Table 1, with included chapters listed alphabetically. Citations are provided for these books, but only the identified chapters were counted as sources. Three assessment manuals, four doctoral theses, three professional magazine articles, one professional document and three web sources were included, and eight conference submissions (paper or workshop presentations or posters). No sources included funding data.

In Table 1, sources classified as ‘OT’ are relevant to the profession broadly (38/102). Mental health was the largest practice area represented (43/102) and included sources relating to specific diagnoses or settings, as well as chapters reflecting core occupational therapy processes or skills in books about mental health practice. Other practice areas included cognitive rehabilitation, child/youth, older adult and physical rehabilitation. Half (52/102) presented theoretical content (e.g., presenting models or approaches for practice), with just over one third (38/102) presenting applied content (e.g., descriptions of occupational therapy practice) and the remainder (12/102) representing research and evaluation.

Four broad source objectives were apparent, as seen in Table 1. Only 12 sources had a specific objective of describing formulation or conceptualisation in occupational therapy, with some presenting the use of psychological formulation. The other main objectives included the presentation of MOHO (as a conceptual practice model, framework for practice, tools/assessments and use in practice; 41/102); description of the occupational therapy practice process or theory‐practice links unrelated to MOHO (30/102); and occupational therapy interventions or research within specific practice contexts (19/102).

### 3.2. Development of Occupational Formulation

The history of occupational formulation is considered in only three sources [103, 110, 114], which described the development of case formulation from the mid twentieth century within psychotherapy and psychology, to a place of prominence within mental health settings and its more recent use in medicine, nursing, social work and occupational therapy. The narrative synthesis of sources in this review identified four concurrent development strands of occupational formulation. The first strand is represented in publications associated with MOHO, and the second strand in chapters describing the occupational therapy practice process. The third and fourth strands, respectively, identified occupational therapists′ use of psychological approaches to formulation combined with occupational concepts, and bespoke occupational approaches to formulation. Years, terms and definitions are bolded to highlight shifts over time.

#### 3.2.1. Strand 1: Formulation/Conceptualization in Publications Associated With MOHO

This strand includes many co‐authored sources with strong representation from the United States and United Kingdom. The **2002** textbook *A Model of Human Occupation: Theory and Application (3rd edition)* [27] is the earliest source referring to the target concept, with chapters by Kielhofner and Forsyth [28, 35] presenting it as an integral part of the therapeutic reasoning process. Positioned between the phases of collecting information about the person’s occupational life and developing decisions for action and implementing therapeutic goals and strategies, the phase was variously labelled as: ‘develop **explanation of client circumstances** based on client information and theory’(p.163); [35] or ‘create a **conceptualization of the client′s situation** that includes client strengths and weaknesses’(p.326) [28], with an expanded description that ‘the **conceptualization of the client′s situation represents a synthesis of the general concepts of the theory with the particular information about the client**’(p. 326) [28]. Detailed questions for gathering information about occupational identity and competence, volition, habituation, performance capacity, and environment were provided [35]. Although theoretical concepts of occupational identity, occupational competence and occupational narratives; and the importance of client‐centredness and collaborating with the person were identified as important parts of therapeutic reasoning [28, 35], no explicit connections were made between these concepts and the phase of conceptualizing the person’s situation. The importance of documenting was noted [28], but not illustrated within the example of a therapist developing an unstructured conceptualization including concepts of volition, skills and roles. A separate diagrammatic example (p. 165) [35], focused only on concepts of environment and volition. Other chapters mentioned or exemplified use of the approach using stem + focus terms: **conceptualization/theory-based understanding/theory-based explanation *of* the client′s situation/circumstances/strengths and weaknesses**, [29–34] with the standalone terms **conceptualization** [34, 35] and **formulation** [30, 35] used less frequently.

Publications in **2006** described the approach as **case formulation** [39, 40]. Chapters by Kielhofner and Forsyth in the **2008** fourth edition of the MOHO text [49] offered a reworded definition: **‘Conceptualizing the client′s situation requires synthesis of information collected with MOHO concepts to create a unique explanation or understanding of each client’** (p. 411) [51]. They asserted that ‘there are many ways to develop your conceptualization of a client′s situation’ (p.415) [51], while also stating that use of a structured process can help with organising thoughts, making connections aligned with theory and identifying information gaps. However, instead of a structure, they offered an extensive list of reflective questions to guide development of a conceptualization, encompassing assessments, occupational identity, occupational competence, person factors (personal causation, values, interests, roles, habits, performance capacity) that may prevent or support occupational engagement; the contribution of environmental factors and a conclusion statement [51]. The importance of using feedback to share the conceptualization with the client was emphasised [55], and the need to document it [51] illustrated with two examples, each using different headings linked to MOHO concepts. Other chapters mentioned or exemplified use of conceptualization [52, 53, 148].

Parkinson and et al.′s **2008** case study [57] used information gathered via the Model of Human Occupation Screening Tool (MOHOST) [39] to develop a **case formulation** in an acute psychiatric setting. Emphasising the importance of ‘explaining the client′s circumstances rather than simply giving a list of performance strengths and limitations’ (p. 69), clear links to MOHO theory were made, with explanation and demonstration of how the client’s circumstances (including strengths and limitations) could be presented using structured sections: occupational identity, occupational competence and key issues preventing or supporting occupational adaptation.

Between **2009** and **2017**, a range of terms were used to identify the approach, with **occupational case formulation** appearing in Parkinson′s work from 2011 [71, 72, 92, 95] and **occupational formulation** used in a **2014** chapter about MOHO [82] and extensively in the **2015** ACHIEVE manual [84]. O′Brien et al. [65] presented a six‐step therapeutic reasoning process to guide occupational therapy input with children, which included detailed examples of written **conceptualizations of the client′s situation** structured with headings of volition, habituation, performance capacity and environment. Similar descriptions appeared in CIRCLE [58] and ACHIEVE paediatric assessment manuals: **‘integration of all these perspectives [of child, parents, school, OT] allows the practitioner to develop a realistic and comprehensive account of a young person’s occupational life…referred to as occupational formulation’** (p. 2) [84].

Forsyth′s chapter [101] in the **2017** fifth edition of the MOHO textbook defined the approach: **‘Occupational formulation describes…how an occupational therapist takes all the assessment information for a client and pulls it together in order to create a set of arguments about their unique perspective of their client′s occupational situation’** (p. 164) [101]. Reflective questions indicated the need to explore the person′s and therapist′s views on identity, competence, positive participatory issues and participation challenges, to identify three key participatory issues. Again, no structure was articulated, but a very brief example outlined the roles, values, limitations and intentions of a man hospitalised for the treatment of depression. Other chapters [99, 100, 102] used different terms when mentioning or exemplifying this step of the therapeutic reasoning process, including labelling it **clinical hypothesis** [98], rather than building directly on Forsyth′s chapter.

Brooks and Parkinson′s **2018** opinion piece [103] described **‘occupational formulation is a process of making sense of someone′s circumstances that is informed by occupational theories and concepts…a stage in the occupational therapy process that emerges from professional reasoning’** (p. 177). Seeking to address the lack of common approach, they proposed the use of three‐part occupational formulations presented in narrative form with explicit connection to a model. To support compatibility with any occupational therapy model, the three parts could be described as occupational influences, occupational presentation and occupational focus. They described that when using MOHO, the parts could be named occupational identity, occupational competence and key occupational issues. A structured example was presented.

Building on their previous work, Parkinson and Brook′s **2021** guidebook [110] detailed the history and benefits of the approach, noting: **‘occupational formulation is a reflective process that synthesises rich information and requires in-depth occupational therapy assessment. It will draw upon the values and goals of the person, their appraisal of their own ability, and their expectation of success in the future’** (pp. 8–9). This book offered detailed guidance for developing MOHO‐based occupational formulations, in which the occupational identity section communicates the person′s subjective perspective and the occupational competence section describes the occupational therapist′s perspective before agreement of key occupational issues for occupational adaptation. Three processes by which an occupational formulation communicates clinical reasoning are described: collaboration, contextualisation, and conceptualisation. The first was consistent with MOHO textbook descriptions about the importance of collaborating with the person when developing the occupational formulation. The second reflected the importance of integrating elements such as the person′s environment and history. The third process implied that conceptualization is part of, but not synonymous with formulation: ‘Conceptualising complex needs to establish a case for intervention’ (p. 9). Twenty detailed examples of occupational formulations from a range of practice areas were provided. Since **2020**, other authors [107, 109, 111, 112, 119] have cited the work of Forsyth [133], Parkinson and Brooks [103, 110] when describing MOHO‐based occupational formulation.

Descriptions within the **2024** MOHO textbook [128] returned to use of the term **conceptualization** and the focus on strengths and challenges of earlier editions. The main chapter presenting the therapeutic reasoning process [132] emphasised the client’s occupational narrative along with the need to consider subjective elements of occupational participation, with inclusion of objective elements implied. Although clear links between MOHO concepts and the structure or content of the **conceptualization of strengths and challenges** were lacking, prompts were provided to consider volition, habituation and performance capacity within the context of the environment with the aim of understanding occupational adaptation. This approach is suggested as a basis for hypothesis development: ‘The combination of the client’s narrative and occupational strengths and occupational challenges leads to an initial conceptualization of the client in relation to the MOHO concepts. Short and long‐term goals are built upon the hypotheses generated from this conceptualisation’ (p. 186). Another chapter [129] appeared to conflate these processes when describing the step of conceptualization, focusing on the synthesis of information to create hypotheses. Other chapters mentioned or exemplified use of **conceptualization** [129–131], and introduced the additional term **case insights and decisions** [131].

#### 3.2.2. Strand 2: Formulation Within the Practice Process

A second strand in the development of occupational formulation was identified in chapters by UK authors presenting or exemplifying the occupational therapy process. Creek′s **2003** report, *Occupational Therapy Defined as a Complex Intervention*, [36], described an 11‐step occupational therapy practice process. The fourth step ‘reason for intervention; **needs identification**; **problem formulation**’ followed initial assessment and preceded goal setting. The importance of collaboration throughout the practice process and the need to use one or more theoretical frameworks to support problem formulation were emphasised.

Between **2006** and **2021** subsequent sources (many in sequential editions of key textbooks) referred to Creek′s practice process [38, 43–45, 70, 79, 81, 106], offering slightly different emphases and descriptions of the formulation step, such as obtaining ‘**a picture of the client′s overall functional ability and problem areas so that the need for intervention can be established**’ (p. 83) [44]. The interchange of key search terms was apparent: ‘Taking time to **formulate** all the information gathered through the assessment process into a **conceptualisation** of the individual is an important step in the assessment process and vital to enable appropriate planning of an intervention. **The conceptualisation, drawn from the information gathered, will provide a portrait of a client′s occupational life and their needs. This information should be considered as a whole**’ (p. 81) [60]. In one chapter the term **collating assessment findings** was used to describe the organisation of ‘occupational needs, occupational skills and occupational performance’ into strengths, skills and problems [81].

In the **2022** sixth edition of *Creek′s Occupational Therapy and Mental Health* [115], a simplified description of the ‘occupational therapy process’ included ‘**formulation** and expected outcomes’ with a focus on promoting understanding, informing goal setting and communicating plans. One chapter [116] mentioned **case formulation** to support intervention planning and another described ‘occupational formulation’ as a ‘**systematic way of explaining the difficulties that an individual is currently encountering and how these impact their occupational functioning**’ (p. 100) [114]. It also included a description and practice example based on Parkinson and Brook′s [103] universal approach.

#### 3.2.3. Strand 3: Formulation Approaches Drawn From Psychology

The third development strand, with all but one source originating from the United Kingdom, focused on occupational therapists′ use of psychological approaches to formulation, with addition or integration of occupational concepts.

Writing by Duncan [149–153] offered important context for a cluster of sources. He described the use of cognitive behavioural theory to complement occupational therapy models, often including cognitive behavioural **case formulation** of the client′s problems as a working hypothesis to communicate the therapist′s perspective and to invite clients′ collaboration to refine and use it in therapy. Appearing to build on this foundation, between **2008** and **2017,** Duncan and others in a range of mental health settings described or demonstrated the use of occupational therapy assessments or concepts (most MOHO‐based) to inform their cognitive behavioural formulations [47, 48, 80, 89, 96].

Other uses of psychological approaches to formulation included a **2004** report about life skills training for people diagnosed with schizophrenia. It presented a bespoke approach to formulation of functional difficulties, without clear links to occupational therapy theory, but using the stress vulnerability model together with considering developmental, social/environmental/sensory‐motor and psychological factors [37]. Between **2010 and 2015,** several authors devised approaches that combined MOHO concepts with psychological‐based approaches to offer occupationally based **case formulations** [85] or **occupational formulations** [66, 75, 77] to the multidisciplinary team (MDT). Approaches focused on understanding offending behaviour and risk in forensic settings combined core MOHO concepts with offence‐paralleling behaviour and the Good Lives Model [66], or multiple sequential functional analysis formulation [85]. In a UK brain injury service, occupational therapists mapped occupational identity and occupational competence onto the Y‐shaped model of rehabilitation [75], and depicted an explanatory diagrammatic formulation with occupational identity at its centre within the narrative of a survivor′s story [77].

Advocating the use of **case formulation** in complex occupational therapy presentations, New Zealander Thompson [4] presented an approach in **2012** based on a *process* of formulation described in clinical psychology [154]. Using abductive reasoning to work ‘from descriptions of patterns to possible explanations of those patterns’ (p. 18), generate hypotheses, then conduct clinical experiments to confirm hypotheses, the five steps included detecting the underlying patterns, inferring causal mechanisms, developing a causal model, evaluating the causal model and writing the case formulation. The importance of documentation was emphasised but not demonstrated within the presented scenario. An argument was made for using formulation in occupational therapy: to address the complexities of the presentations seen, the need to move beyond diagnoses to understand the person′s experience, the potential presence of multiple diagnoses and to support tailoring of interventions to the person.

#### 3.2.4. Strand 4: Bespoke Occupational Approaches to Formulation

Most sources in this strand presented occupation‐based formulation approaches that appeared to have been developed by practising occupational therapists independently, and without reference to other published work about formulation in occupational therapy. Nevertheless, several drew on models of practice. For example, in a **2011** blog post about hoarding, Dora [69] proposed an approach to assessment and **case formulation** that could ‘**provide an occupationally focused framework for organising and analysing information from assessments to identify OT-specific goals and interventions**,’ providing detailed prompts for consideration of MOHO concepts. Between **2012 and 2017**, others described use of MOHO to create **occupational formulations** in mental health and intellectual disability settings [74, 76, 90], and one chapter [89] presented an example informed by MOHO assessments and structured with headings that appeared to be drawn from the Canadian Model of Occupational Performance [155].

Having observed psychologists creating formulations within an NHS mental health service, Bullock [73] presented an **occupational formulation** approach based on the activities health model [156] at a **2012** conference. Emphasising collaboration to determine needs, set goals and plan therapeutic activities, a diagrammatic structure was used to document the meaning and relevance of activities for the person, assessment results about their condition and its effects on performance components, and their strengths and difficulties. A **2016** thesis by Brooks [87] described child and youth mental health occupational therapists being expected to bring **occupational formulations** (short narratives about occupational participation and engagement in relation to problems with everyday activities) to contribute to the broader formulation of the MDT.

Inman′s [91] **2017** doctoral thesis about occupational therapy for people living in the community with psychosis reported development of an occupational therapy pathway (involving critique of an existing intervention schedule, review of practice guidelines from an NHS trust and expert consultation) with **occupational needs formulation** following assessment of occupational performance and preceding setting occupational need goals. She indicated any model of occupational therapy could be used when formulating occupational needs, with the process involving four key activities to: ‘describe the individual′s occupational performance in the participation of their activities of everyday life, identify with the individual potential barriers to optimum occupational performance, identify the individual′s occupational performance abilities (strengths) which could be used to help minimise and/or overcome barriers to participation of their activities of everyday life and discuss and agree occupational needs formulation with the individual (and carer as appropriate)’ (p. 364).

In **2019** and **2025**, chapters in sequential editions of the North American text *Occupational Therapy in Mental Health: A Vision for Participation* [147, 157] focused on the PEO model [105, 137] described synthesising understandings of assessment information into a **formulation of occupational performance issues**, which identified the supports/constraints for occupational participation in an identified occupation within a specific environment. Client collaboration was emphasised as important for (in)validating and elaborating upon the formulation and identifying options for improving PEO fit.

Identified in **2025**, the website of a US private occupational therapy practice was the only source providing information about occupational formulation for the public/potential clients of occupational therapy. It described **occupational formulation** as ‘the secret sauce’ to ‘ensure therapy is aligned with what truly matters to the client.’ Using core MOHO domains of volition, habituation, performance capacity and environment to guide the process, occupational formulation was described as ‘a **structured way of thinking about a person′s needs, challenges and strengths as they relate to meaningful daily activities (occupations). It is a tool occupational therapists use to understand and explain why someone may be having difficulty participating in life the way they want to**.’

#### 3.2.5. Terms Used

As reflected in Table 1 and the development strands, a range of terms beyond the initial search terms were used, often with multiple terms interchanged within one source or across chapters in an edited book. Other terms not covered in the table and development strands include identify occupational challenges [121, 122], setting the problem/problem‐setting [56] and creating a picture of the person′s experience [64].

### 3.3. Features of Occupational Formulation

Key features of occupational formulation were rarely articulated explicitly but were reflected in descriptions of the approach and its use. Six key features were identified, as reflected in Table 1.

#### 3.3.1. Incorporates Theory, Model or Framework

The need to link occupational formulation to a theory, model or framework was identified in more than three quarters of sources. Although several identified the need for a model or framework/s without adopting or citing one [45, 79, 114, 115, 117, 36], the majority referred to use of MOHO for developing a formulation, some combined its use with another model or framework [47, 66, 73, 75, 80, 85, 89, 105], and two referred to use of PEO [105, 137].

#### 3.3.2. Sits Within Practice/Reasoning Process

More than half the sources situated occupational formulation within the occupational therapy practice/reasoning process, between phases of assessment and goal development/intervention planning. Some provided broad phase descriptions unlinked to a recognised practice process [69, 73, 91], whereas most linked to the MOHO therapeutic reasoning process or Creek′s practice process.

#### 3.3.3. Part of a Collaborative Process

One third of sources described working in a client‐centred or collaborative way. Some noted the importance of collaborating with the person to support engagement [47, 101, 109, 36] and/or emphasised the need to involve caregivers [83] or others [115]. Others offered more detail: The occupational therapist should invite collaboration; [28] seek the person′s perspective [56] through use of thorough occupational assessments [110] which may include conversations [87], self‐assessments [80] and narrative techniques or assessments like the Occupational Performance History Interview (OPHI‐II) [83]. Wherever possible, the occupational formulation should be shared with the person [28, 98, 102, 105, 110, 137], allowing development of a shared understanding and collaborative therapy planning (see further details in purpose of occupational formulation).

#### 3.3.4. Involves a Narrative Approach

Some sources emphasised how the occupational formulation reflected the narrative element of the person′s story, using phrases like recounting lived experience/telling the story/sharing the client narrative/recrafting occupational narratives [83, 95, 103, 110, 112, 118, 119, 132]. Most examples were presented in narrative form and written in third person, with a few exemplifying use of second person [47, 103, 110, 114]. Several mentioned the narrative reasoning of the therapist as important for understanding the person′s situation [58, 72, 73, 134]. Those that encouraged documentation of the occupational formulation also emphasised the need to present the information as a narrative [4, 72, 87, 91, 103, 110, 114, 118].

#### 3.3.5. Has a Structure

Approximately half the sources presented some kind of example occupational formulation, ranging from several sentences [101] to several pages [110]. Although many lacked a clear structure, most reflected use of theoretical concepts and included some information from the person′s perspective. Only the work of Parkinson et al. [57, 72, 92, 95, 103, 110, 127] (and others citing them) outlined a clear structure for documenting an occupational formulation, integrating theoretical concepts and contributions from both the person and therapist.

#### 3.3.6. Involves an Ongoing Process

Several sources emphasised that occupational formulation is an ongoing process/develops over time [47, 87, 91], and may be confirmed or changed during therapy [28, 45, 53, 62, 73, 79, 96, 114, 121, 124, 129, 132], or revised at the conclusion to demonstrate outcomes [84]. Only five sources mentioned both the documentation and potential revision of occupational formulations in practice [28, 73, 87, 91, 114].

### 3.4. Purpose of Occupational Formulation

Reasons for using occupational formulation are presented in the ‘purpose’ section of Table 1 and described here. Five key purposes were evident.

#### 3.4.1. Synthesises Assessment Findings

Using varying language, such as presents, summarises, condenses, organises or analyses, more than a third of sources described that the occupational formulation integrates or synthesises assessment findings. This could include both subjective and objective information, such as self‐reported and historical information, information from key others, observations and findings from structured assessments.

#### 3.4.2. Supports Therapeutic Reasoning

Sources reflected a common view that occupational formulation has value in supporting therapeutic reasoning, making links between theory and the person′s presentation and considering how to move forward. This was exemplified when Brooks and Parkinson′s [103] universal approach was used as a framework for teaching professional reasoning to Thai occupational therapy students [117]. However, descriptions of how occupational formulation supports reasoning often lacked clarity. Some sources mentioned that it allows ‘analysis’ of information and/or leads to ‘explanations’ or ‘hypotheses*’* about what is affecting the person′s occupational participation. Descriptions of hypothesis formation were particularly blurred: Some sources described development of the occupational formulation to provide explanations that could inform hypotheses about a person′s occupational participation; [58, 93, 132] others conflated the concepts by describing the conceptualization as a (clinical) hypothesis [4, 98, 124], or describing the process of hypothesis development when presenting the conceptualization/formulation step in the MOHO therapeutic reasoning process [93, 94, 98, 123, 124, 129].

Contrasting with sources portraying analysis [66, 69, 73, 123], Parkinson and Brooks [110] asserted that occupational formulation is *not* an analysis, and that ‘analysis can always be challenged, whereas the formulation tolerates different perspectives’ (p. 55) and showcases the person′s story, rather than the therapist′s analysis. However, use of analysis is reflected in their guidebook (in commentary about Example IV and in the therapist′s perspective in Box XXI). Additionally, although not writing explicitly about hypothesis formulation when describing how occupational formulations are developed, their presentation of the history and purpose of formulation (drawn from psychological literature) included multiple references to hypotheses, either flowing from or being equivalent to the formulation.

#### 3.4.3. Supports Shared Understanding and Therapy Planning

Sharing the occupation formulation with the person with whom they are working allowed the occupational therapist to share their understanding/perspective [28, 60, 81, 105, 110] and an opportunity to give therapeutic feedback [28, 54, 58, 99], promote awareness and understanding [4, 115]. Some sources suggested the person may validate the accuracy of information [28, 57, 60, 105, 137] and offer their interpretations [80, 98], so that a common perspective/shared understanding can be reached [28, 57, 75, 110]. Half the sources identified that occupational formulation supports the selection of priorities for therapy, informs goal development and supports planning to guide therapy provision. Kielhofner and Forsyth [34] articulated how the conceptualization may inform therapy plans by pointing towards the types of engagement and therapeutic strategies that might support a person′s occupational engagement and change.

#### 3.4.4. Supports Communication

Using occupational formulation supported communication of assessment findings, goals and plans with the wider team [4, 107, 115]. Importantly, it assisted the occupational therapist to articulate their reasoning for use of an occupational focus [4, 51, 72, 73, 95, 103, 110, 119], which could help to remediate the difficulties experienced in communicating their reasoning [73, 110]. Documentation of the occupational formulation is mentioned in 15 sources: to explain the person′s presentation [4, 114] and/or provide a rationale for the goals and therapy approach to the wider team [4, 28, 51]. Two sources described it being recorded as a diagram and shared with the person and/or team [73, 77], whereas others describe or exemplify use of a written record.

#### 3.4.5. Supports an Occupational Focus

Using occupational formulation fostered an occupational focus for clinical reasoning and service delivery [56, 64, 69, 87], an occupational perspective of the client′s circumstances [109, 111], and a stronger understanding of occupational issues/needs [72]. It also assisted in communicating the rationale for an occupational focus [110] and the value of occupational therapy [85, 112]. Although just over 10% of sources articulated how using occupational formulation supports an occupational perspective, we infer that the many authors who linked the use of formulation with occupational therapy theory or models assumed that using a theoretically based formulation approach would support an occupational focus in practice.

### 3.5. Potential Benefits of Occupational Formulation

Parkinson and Brooks [110] proposed four benefits of occupational formulation: first, *benefiting the person and carers* by reflecting their voice and structuring a personalised approach; and second, *enhancing the therapeutic relationship* as collaborative development of the narrative strengthens the relationship and facilitates a shared understanding of issues and meaning. Third, they proposed its benefit in *assisting the therapist* to develop a deeper understanding of the person and of how to address issues, identify information gaps, link theory and assessment information, structure reasoning, prioritise issues and guide intervention planning, leading to increased confidence in their work. *Informing the wider team,* through greater transparency of occupational therapy reasoning, clearer team understanding of therapy goals, and contributing to joint MDT formulations, was proposed as its fourth benefit. These are consistent with benefits described with less clarity elsewhere [4, 73] and reflected in the aforementioned purposes for using occupational formulation.

Several sources by Parkinson and Brooks [71, 92, 95, 110, 118] and others [75, 85, 112] included occupational therapists′ practice reflections on using occupational formulation consistent with the four proposed benefits. However, as shown in Table 1, few sources presented research or evaluations. Only one of these focused specifically on occupational formulation [72], whereas others evaluated the therapy process, including occupational formulation. The four proposed benefits are used below as a framework for synthesising these research accounts.

#### 3.5.1. Benefiting the Person and Carers

Several sources illuminated how use of occupational formulation could benefit clients and carers. Based on a satisfaction survey conducted within an NHS mental health service, Parkinson et al. [72] reported that clients appreciated feeling understood and supported in clarifying their thoughts, priorities and making plans through using occupational formulation and goal setting. Inman′s [91] feasibility study within two community teams in the United Kingdom sought to understand how occupational therapy enabled people diagnosed with schizophrenia to participate in everyday activities. Through information gathered from seven occupational therapists and 16 service users, this study found that 93% of service users expressed satisfaction with how their therapist assisted them to identify what helped or hindered their participation. The study highlighted that occupational needs formulation involved a collaborative, client‐centred process that enabled service users to anticipate, accept and overcome barriers to participation, and acted as a catalyst for insight and change. Using the same occupational therapy intervention protocol as Inman, Christie [108] evaluated the effectiveness of individualised occupational therapy in a UK service to improve occupational functioning and participation in everyday activities for community‐dwelling people with depression. Care records and intervention logs indicated that the component ‘agree occupational formulation’ was completed with all participants. Evaluation showed statistically significant improvements on multiple outcome measures for the 18 participants who completed therapy: Canadian Occupational Performance Measure (COPM), [158] Beck Depression Inventory II and [159] Work and Social Adjustment Scale, [160] two scales of the Ultrecht Scale for Evaluation of Rehabilitation–Participation [161] and three scales of the Short Form‐36 Health Survey [162]. Qualitative findings from interviews with seven service users highlighted that they valued the opportunities for leadership, partnership and coproduction during therapy.

Graff′s [83] chapter summarised a body of research that involved implementation and evaluation of a community occupational therapy programme (including strengths, needs and case formulation phase) for people living with dementia and their carers. A randomised controlled trial with 135 participants found improvements in participants′ daily functioning, mood, quality of life and caregiver sense of competence, and demonstrated the intervention was cost‐effective. In a study involving 10 occupational therapists and 52 service users from one UK mental health service, Parkinson et al. [86] reported that service users working with therapists in profession‐specific roles, where there was the opportunity to use occupational formulation, had significantly higher satisfaction in a wider range of daily living activities than service users working with occupational therapists in non‐profession‐specific roles.

#### 3.5.2. Enhancing the Therapeutic Relationship

Use of occupational formulation enhanced the therapeutic relationship through supporting collaborative working. Occupational therapists in Inman′s study [91] reported that the formulation process ‘enabled us to anticipate, accept and overcome barriers to participation’ (p. 261) and supported agreement of plans between therapists and service users, helping to set priorities and direct the focus of individual sessions. Similarly, in grounded theory research within UK perinatal mental health services, Graham [107, 119] reported that occupational therapists used formulation to support collaboration with service users. Parkinson et al. [72] also noted that clients valued opportunities to clarify their priorities and be actively involved in planning, which likely contributed to stronger therapeutic alliances.

#### 3.5.3. Assisting the Therapist

Using occupational formulation assisted occupational therapists in several ways. The satisfaction survey by Parkinson et al. [72] found that when using occupational formulation and goal setting, occupational therapists reported increased person‐centred practice, a better understanding of occupational issues, improved treatment planning and increased confidence. Similarly, Wimpenny et al.′s [40, 68] participatory action research project sought to embed MOHO assessment and documentation (including occupational case formulations) within a UK mental health service, and resulted in an increased occupational focus [40, 68]. Inman [91] also reported that when ‘occupational needs formulation was the focal point’ (p. 239) of the intervention, it helped therapists anticipate barriers and structure sessions effectively. High fidelity ratings for formulating occupational needs, with 84% adherence captured in therapist logs, also indicate that these therapists valued this process. Brook′s [87] ethnographic research of two occupational therapists working in children and young people′s mental health highlighted that ‘concluding occupational formulations’ were used by therapists to enact occupational practice and resist the dominance of medical and psychological models. O′Brien et al. [65] found that using the MOHO therapeutic reasoning process, including the step of creating a conceptualisation, supported student therapists in delivering individualised, occupation‐based interventions with children. Parkinson et al. [86] found that occupational therapists in profession‐specific roles completed more occupational formulations and found their work environment more satisfying than their counterparts in generalist roles.

#### 3.5.4. Informing the Wider Team

Occupational formulation supported occupational therapists to share their perspectives with colleagues, according to several UK sources. In Graham′s [107, 119] grounded theory study, the use of formulation assisted occupational therapists to articulate their assessment findings to the team, whereas Brooks [87] observed that in doing this, occupational therapists provided a specialist occupational perspective within teams that could complement or challenge dominant team narratives. Connell′s [88] research survey found that forensic occupational therapists contributed to the team by conceptualising offending as an occupation to offer a formulation of reoffending risk from an occupational perspective. Parkinson et al. [72] reported that adopting formulation in an occupational therapy service enhanced team working and promoted the occupational therapy role.

## 4. Discussion

This scoping review has described the emergence of occupational formulation and its use in occupational therapy practice. The review identified some peer‐reviewed literature reporting research or evaluation, with most sources being grey information and most originating from the United Kingdom or United States. Four development strands were traced through the 21st century: within publications associated with MOHO, within chapters about the practice process, approaches drawn from psychology and bespoke occupational approaches. The findings suggest that occupational formulation has been predominantly used in mental health settings. However, its purposes indicate it is more widely applicable in the occupational therapy practice process: to structure findings from assessment; support therapeutic reasoning and practice application of theoretical frameworks; inform goal development and therapy planning; facilitate shared understandings and enhanced communication with clients, carers and colleagues; and contribute an occupational perspective to multidisciplinary teamwork.

The first two development strands highlight the efforts of occupational therapy researchers and educators describing how to link occupational therapy theory and practice processes. In the first strand, this is most evident in textbooks about the MOHO where a conceptualization/formulation step has been included within the therapeutic reasoning process since 2002 [28, 35, 51, 54, 101, 132]. While emphasising connection to MOHO concepts, the description of this step has varied over time within MOHO textbooks, with little clarity provided for how a conceptualization/formulation might be created or documented. In 2017, Forsyth [101] provided the first textbook definition of occupational formulation. Subsequent major development in the description and exemplification of its use with MOHO has been presented by Parkinson and Brooks [103, 110] but is not reflected in the most recent MOHO textbook [128]. Similarly, the second strand focused on the use of formulation in descriptions of the occupational therapy practice process, mostly by UK‐based authors with a mental health background. Sometimes labelled as ‘problem formulation’, this terminology suggests that this use of formulation may have developed from Hagedorn′s [163] work that described naming/defining/identifying the origin of/framing problems. Overall, little guidance was provided for enacting this step.

The third and fourth strands reflect the efforts of practising occupational therapists creating formulations either by adding an occupational perspective to psychologically based conceptual or process frameworks or by creating bespoke occupational approaches to formulation for specific practice settings. Many of these authors drew on MOHO concepts when creating formulations, which may reflect widespread use of MOHO in UK mental health settings [164] and/or its broad suite of assessment tools for use in practice. However, awareness of published descriptions of MOHO‐based formulation/conceptualization was not evident in their work.

Much of the initial development and use of occupational formulation has been led by occupational therapists working in mental health services. Their exposure to other forms of formulation (often psychological) likely inspired efforts to create profession‐specific formulations. That many sources were UK‐based may reflect the widespread involvement of occupational therapists in providing mental health care, and the influence of various scholarship of practice projects to embed MOHO and/or occupational formulation within mental health services [68, 72, 165–167]. Nevertheless, since its introduction in 2002, formulation or conceptualization has been consistently identified as a step in the MOHO therapeutic reasoning process and in the occupational therapy practice process informed by Creek′s work and described in UK textbook sources. As such, it holds relevance for occupational therapists in wide‐ranging practice areas to support them in making connections between occupation‐based assessment and identification of priorities, development of goals, planning and delivering therapy, as reflected by the examples of its use in varying settings within Parkinson and Brook′s [110] guidebook. Brooks and Parkinson [103] noted potential synergies between their occupational formulation approach and the Occupational Therapy Practice Framework [168]. To date, it is not articulated in this nor other practice process frameworks, such as the Occupational Therapy Intervention Process Model [169] and Canadian Occupational Therapy Inter‐Relational Practice Process [170].

Consistent with Joosten′s [171] call for use of occupational therapy conceptual models to be demonstrated to ensure occupation‐centred practice, many sources emphasised that a formulation must be linked to a model or framework, supporting both an occupational focus and connection between theory and practice. Few sources in this review provided clear guidance for *how* to link formulation to a model, although most MOHO textbook sources emphasised a focus on strengths and challenges and/or varied MOHO theoretical concepts when developing a formulation, potentially leading to long and disjointed formulations. Parkinson and Brook′s [103] three‐part structure offered an approach using universal headings (occupational influences, occupational presentation and occupational focus) compatible with any occupation‐focused model, or using headings linked to the three highest level MOHO concepts: occupational identity, occupational competence and (key issues for) occupational adaptation. Serving as a knowledge tool or product [172], the existence of a clear structure such as this is likely to decrease the translation gap for therapists seeking to use occupational formulation in practice.

The description of formulation as both a process and a product [173] is useful when considering the emphasis of different sources. Many sources emphasised the importance and benefits of the *process* of developing an occupational formulation, leaving readers unclear about *how* to implement this process in practice, as experienced by Jamieson and Parkinson [92] when looking to the 2008 MOHO textbook for guidance. More often implied than made explicit, many descriptions suggested formulation is largely a reasoning process, occurring within the mind of the occupational therapist. Fewer sources emphasised the creation of a *product* or written occupational formulation to support articulation of the occupational therapist′s reasoning, and to enhance communication with the person and the team through sharing the document or a verbal summary. A written formulation may offer additional benefits to support the person′s recall, their understanding of information discussed or therapy activities, and may be therapeutic in itself [174].As D′Cruz et al. [174–176] reported in their research involving people with acquired brain injury, written or recorded narratives provided opportunities for validation, processing feelings, losses and challenging situations, and building a strength‐based identity.

The use of narratives and narrative reasoning by occupational therapists has previously been identified as a central mode of occupational therapy reasoning, involving narrative talk and story creation [177]. Narrative talk is a mode of speech used when occupational therapists tell stories about their clients, reflecting understanding of the person′s experience and life situation. Story creation, or therapeutic emplotment, is how therapists create a therapeutic story to meaningfully connect therapy with the person′s larger narrative. The use of narrative in occupational formulation may foster both types of narrative thinking. Documenting the formulation is a form of storytelling that goes beyond a vocalised narrative about a person to conscious inclusion of content from their perspective in a shared, written narrative. In turn, by collaboratively identifying key occupational issues/needs, goals and therapy plans, occupational therapists invite people with whom they work to create a shared story or therapy plot. Chapters in MOHO textbook editions since 2002 have presented the use of occupational narratives [148, 178–180], defining these as stories told or enacted, in which plots and metaphors bring together elements of volition, habituation, performance capacity and environment to reflect a person′s evolving occupational identity, occupational competence and ultimately, their occupational adaptation. Additionally, chapters about recrafting occupational narratives [50, 100, 181] have provided examples of using the OPHI‐II [182] (which includes use of occupational identity and occupational competence scales) to develop a storied understanding of the person′s occupational participation over time. Thus, the use of an occupational formulation narrative that is structured by occupational identity and occupational competence concepts [103, 110] is consistent with well‐established use of narrative for applying MOHO concepts in practice.

Sources in this review often highlighted the opportunity and imperative for collaboration in occupational formulation through the sharing of client and therapist perspectives, to negotiate shared approaches for therapy. Framed as client‐centredness in MOHO‐based sources since 2002, seeking the person′s perspective and working together has long been emphasised. Collaborative development of the occupational formulation and goals also reflects a commitment in occupational therapy to power‐sharing principles [183]. Yet, several sources highlighted that occupational formulation should include both subjective information from the person and objective information from the therapist. Use of the terms *subjective* and *objective* to label sections or types of information is inconsistent with a truly collaborative approach to therapy and may inadvertently devalue the person’s perspective while privileging the occupational therapist′s view [184].

### 4.1. Proposing a Term and Concept Definition

Few definitions of occupational formulation exist and a lack of consistency and clarity in descriptions was evident in the reviewed sources. Forsyth [101] published the first definition of ‘occupational formulation’ in 2017, and Parkinson and Brooks provided further conceptual detail and guidance for its implementation in 2018 and 2021. Nevertheless, perhaps reflecting regional language preferences, the term ‘conceptualization’ appears to be favoured in sources from the United States, and ‘occupational formulation’ in the United Kingdom and elsewhere. Similarly, in psychology literature, ‘case formulation’ and ‘case conceptualization’ are sometimes seen as equivalent terms [11, 15]. Yet, aligned with their distinct dictionary definitions [16], Parkinson and Brooks [110] described conceptualization as a process *within* formulation, implying that the terms are not interchangeable. Given the term *conceptualization* is frequently used in occupational therapy literature to describe developing an idea in a general sense, we propose that the term *occupational formulation* be adopted when referring to this aspect of the occupational therapy practice process, for three reasons:

1. Inclusion of the adjective *occupational* communicates the focus of our profession.
2. The term *formulation* more precisely reflects both a structured process and the creation of a structured document.
3. The term *occupational formulation* has been developed and used in association with and separately from practice applications of MOHO.

Use of the term *occupational formulation* does not imply a one‐size‐fits‐all approach to using theory in practice; rather, it is the overall process and the structure of the final product that follow a formula: a collaborative process of integrating information about a person′s occupational participation and creating a document structured using theory‐based concepts.

It is important to clarify *what* is being formulated. Although the reviewed sources reflect use of an occupational lens to develop an understanding of the person′s occupational participation and challenges, there is inconsistency in how this is described as problems, issues, needs, circumstances, strengths and weaknesses, an explanation or understanding, or a course of action. Psychological approaches often start with a problem to be formulated, working ‘backwards’ to identify contributing factors; or with a hypothesis to guide seeking evidence to support or refine it. The reviewed sources generally indicated that occupational approaches to formulation operate in a ‘forward’ direction, seeking a holistic understanding of a person’s occupational participation to identify what could be addressed to enhance occupational participation and engagement. Hence, it is *an understanding of a person′s occupational participation and needs* that is being formulated.

Based on our synthesis of reviewed sources, we propose brief and detailed definitions of occupational formulation to support its consistent use in research and practice:

(Brief) Occupational formulation is a theory‐based synthesis of assessment information to understand a person′s occupational participation and needs.

(Detailed) Occupational formulation supports therapeutic reasoning. A step in the occupational therapy practice process, it is both a process and product. Developed collaboratively with a person, it involves synthesis of assessment information with theoretical concepts from an occupational therapy framework or model to reach a shared understanding of a person′s occupational participation and needs. This informs selection of priorities, goals and plans for therapy. The resulting occupational formulation is a structured, written narrative that communicates an occupational perspective and reflects the person’s views and the occupational therapist′s reasoning.

### 4.2. Opportunities for Conceptual Development

Further conceptual development regarding the place of analysis, explanation and hypothesis in relation to occupational formulation would be helpful, given apparent lack of clarity in their use. In the process of developing an occupational formulation, assessment findings are analysed alongside theoretical concepts and then synthesised into a coherent whole. Hence the processes of analysing sources of information and then synthesising them are complementary and interconnected. Parkinson and Brooks [110] have suggested that analysis should be avoided in occupational formulation, whereas it seems likely their concern is more about the inclusion of hypotheses. In occupational therapy, hypothesis generation may be described as part of the assessment process to guide the focus and tools used in information‐gathering [168]. In this sense, the use of hypotheses can be important in generating assessment findings, but their role in occupational formulation is less clear, particularly where the intent is to develop formulations collaboratively with clients.

Authors of reviewed sources made efforts to link information about the person to theory to develop insights into their occupational participation, although they may have drawn upon different types of clinical reasoning. For instance, occupational therapists′ use of procedural reasoning would likely lead to the formation of hypotheses, whereas an emphasis on collaborating with the person to understand their story would draw on interactive and narrative reasoning [1, 2, 185]. Hence, it will not only be important to clarify the role of hypothesis within or following use of occupational formulation, but also how different types of clinical reasoning contribute to occupational formulation in occupational therapy practice. Echoing Brooks and Parkinson′s [103] call for further development and debate, consideration should also be given to potential synergies between occupational formulation and widely recognised conceptual models and frameworks such as CanMOP [170] and the Occupational Therapy Practice Framework [168].

### 4.3. Potential to Strengthen Practice

Occupational formulation may help to address the challenge of making hidden reasoning visible in practice [3]. It offers a framework for linking elements of the practice process and for connecting person‐specific information with theory‐based concepts, which supports occupational therapists′ use of a structured approach to articulate and document their unique perspective. The use of narrative may help to communicate the person′s story and to present the occupational formulation document in a compelling way. Importantly, occupational formulation requires collaboration to present the person′s perspective alongside the therapists′, to promote joint decision‐making, and to ensure therapy is not only tailored to individual needs and preferences but also updated over time.

Occupational formulation holds potential to enhance occupational therapy practice and to support the learning of student occupational therapists through offering a collaborative, occupation‐focused framework for connecting theory and practice [171]. It should not be viewed as additional for busy therapists with already challenging workloads, but rather as a framework to structure practice to address the explicit expectations of professional bodies [5–8] and deliver occupation‐focused and/or collaborative care.

### 4.4. Implications for Future Research

There is a strong theoretical base for occupational formulation, but this review shows research evidence is currently limited. Some support was identified for the four benefits of occupational formulation proposed by Parkinson and Brooks [110], but further empirical studies are needed to establish its acceptability with diverse populations and its effectiveness in different practice settings. Exploring the perspectives of occupational therapists and the people with whom they work will be important to understand its impacts on the quality and outcome of occupational therapy services.

### 4.5. Scoping Review Strengths and Limitations

The strengths of this review lie in its multipart methodology which synthesised peer reviewed and grey sources. Development of the design in accordance with best practice guidelines [19–22] and uploading the protocol publicly prior to commencement supported trustworthiness. Input from an expert librarian, multiple search strategies and detailed reporting strengthen its rigour. However, it is possible that relevant sources were not identified: The ETHOS thesis repository was not searched as planned due to a prolonged technical issue at the British Library, if books were not indexed in Google Books or search terms were not indexed within hard copy books or if potential sources described similar concepts without using the key search terms. Although the inclusion of grey information reflects current practice and discourse [186], critical appraisal was not possible as few sources represented research evidence. As recommended by Levac et al. [20], a formal expert consultation phase was included in the protocol. However, this was replaced by informal consultation integrated throughout the review since the first author had regular opportunities to interact with identified occupational formulation experts, which allowed iterative refinement of ideas. Trustworthiness was enhanced by maintaining a detailed audit trail, and by regular discussions with all review authors regarding selection of sources, extraction, presentation and synthesis of the findings.

## 5. Conclusion

This rigorous scoping review identified a wide range of peer‐reviewed and grey information providing insight into occupational formulation. Its development, features, purpose and evidence were synthesised from these sources. Use of consistent terminology is advocated, and brief and detailed definitions are proposed. Occupational formulation is both a process and a product that may support occupational therapy practice by linking theory to practice, enhancing therapeutic reasoning, ensuring an occupational focus and promoting collaborative care. There is scope to refine occupational formulation, strengthen its empirical foundation and advance knowledge and use of it within occupational therapy.

## Author Contributions

Study concept and design: all authors. Data search and data collection: Lorrae Mynard. Data screening: Lorrae Mynard and Louise Farnworth. Data extraction and analysis: Lorrae Mynard. Data synthesis: Lorrae Mynard and Ellie Fossey. Drafting of the manuscript: Lorrae Mynard. Revision: all authors.

## Funding

Lorrae Mynard was supported to undertake this review through an Australian Government Research Training Program Scholarship. Open access publishing facilitated by Monash University, as part of the Wiley ‐ Monash University agreement via the Council of Australasian University Librarians.

## Disclosure

Final approval of the manuscript was provided by all authors. All authors agree to be accountable for the research presented.

## Conflicts of Interest

Lorrae Mynard is the founder of http://occupationalformulation.com, which provides occupational formulation training. Work on this review commenced prior to the business being founded in response to training requests. Other authors have no conflicts of interest.

## Supporting information


**Supporting Information** Additional supporting information can be found online in the Supporting Information section. File S1: Full search strategies and extraction fields.

## Data Availability

The data that support the findings of this study are available from the corresponding author upon reasonable request.
