# Supplementary material for: Occupational Formulation: A Scoping Review of Its Development and Use
Source: Occup Ther Int. 2026 Jun 19;2026:2011857. doi: 10.1155/oti/2011857 (PMC13282183; doi:10.1155/oti/2011857)
Supplement: Supplementary file 1 — Supporting Information Additional supporting information can be found online in the Supporting Information section. File S1: Full search strategies and extraction fields. [file OTI-2026-2011857-s001.docx]

# Full search strategies and extraction fields

## Full search strategies

**Ovid Emcare** (database records 1995-present), **OVID AMED** (database records 1985-present), OVID **APA PsycInfo** (database records 1806-present) searches conducted 20 Nov 2023 and updated 16 July 2025 using search strategy:

1. Occupational therap*.mp.

2. Formulation.mp.

3. Conceptuali#ation.mp.

4. 2 or 3

5. 1 and 4

**EBSCOhost CINAHL Complete** (1984-present) search conducted 20 Nov 2023 and updated 16 July 2025 using search strategy:

1. Occupational therap*

2. Formulation or conceptualization or conceptualisation

3. 1 and 2

**Google Scholar Advanced Search** conducted 20 Nov 2023 and updated 16 July 2025 using search strategy:

With at least one of the words, anywhere in the article, no date limits:

“occupational formulation”

“occupational case formulation”

“occupational conceptualisation”

“occupational conceptualization”

“occupational case conceptualisation”

“occupational case conceptualization”

**ProQuest Dissertations & Theses Global** (fulltext database records 1997 – present) – Advanced search conducted Nov 28 2023 and updated 16 July 2025 using search strategy:

Noft(“occupational therapy”) AND subject(formulation) OR subject(conceptuali?ation), in dissertations & theses

**Google Books search** conducted Nov 28 2023 and updated 17 July 2025

“occupational therapy” AND formulation OR conceptualisation OR conceptualization

**All internet searches conducted Dec 5, 2023 and updated 21 July 2025**

**Google Search 1**

“occupational formulation”

**Google Search 2**

Occupational formulation

**Advanced Google Search**

“occupational therapy” as “exact word/phrase” and formulation OR conceptualisation OR conceptualization as “any of these words”

**DuckDuckGo Search**

Occupational formulation

**Search within MOHO-IRM website**

<https://moho-irm.uic.edu/>

Archived listserv discussion

Set to earliest date: Jan 1 2000, up to present

Title field search: “formulation”

## Full list of extraction fields

Country

Practice area

Source type

Content type

Source objective

Terminology used

Definition/description provided

History/development of occupational formulation

Purpose of using occupational formulation

Link to practice process

Approach to using occupational formulation

Ongoing/revision process

Documented process

Model/framework referenced

Citations to other selected sources

Reported outcomes of use

Example provided

Other relevant information
